# Perspectives on Admissions and Care for Residents With Opioid Use Disorder in Skilled Nursing Facilities

**DOI:** 10.1001/jamanetworkopen.2023.54746

**Published:** 2024-02-05

**Authors:** Patience Moyo, Shivani Nishar, Charlotte Merrick, Nicholas Streltzov, Emmanuella Asiedu, Corinne Roma, Rahul Vanjani, Jon Soske

**Affiliations:** 1 Department of Health Services, Policy, and Practice, Brown University School of Public Health, Providence, Rhode Island; 2 Warren Alpert Medical School of Brown University, Providence, Rhode Island; 3 Amos House, Providence, Rhode Island; 4 Division of Addiction Medicine, Rhode Island Hospital, Providence, Rhode Island

## Abstract

**Importance:**

Skilled nursing facilities (SNFs) are being referred more individuals with opioid use disorder (OUD), even when their medical needs are not directly associated with OUD.

**Objective:**

To characterize factors that influence SNF admission for individuals with OUD and identify strategies for providing medications for OUD (MOUD) in SNFs.

**Design, Setting, and Participants:**

In this semistructured qualitative study, interviews were conducted with SNF administrators from 27 SNFs in Rhode Island from November 5, 2021, to April 27, 2022. Data analysis occurred from August 22, 2022, to May 31, 2023.

**Main Outcomes and Measures:**

Themes and subthemes on administrator perspectives on admissions and care for people with OUD in SNFs. Audio interviews were transcribed, coded, and analyzed using codebook thematic analysis and guided by community-engaged and participatory research principles.

**Results:**

The study included 29 participants representing 27 SNFs in Rhode Island. Participant roles were administrators (17 participants [59%]), directors of nursing (6 participants [21%]), directors of admissions (5 participants [17%]), and unit managers (1 participant [3%]). Participants described active substance use, Medicaid insurance, housing instability, and younger age as potential barriers to SNF admission for individuals with OUD. The lack of formal guidelines for OUD management, staff shortages, facility liability, state regulations, and skills and training deficits among staff were cited among challenges of effectively meeting the needs of residents with OUD. Many participants reported inadequate institutional capacity as a source of negative outcomes for people with OUD yet expressed their concerns by characterizing individuals with OUD as potentially violent, nonadherent, or likely to bring undesirable elements into facilities. Participants also shared strategies they used to better serve residents with OUD, including providing transportation to support group meetings in the community, delivery in advance of resident arrival of predosed methadone, and telemedicine through the state’s hotline to prescribe buprenorphine.

**Conclusions and Relevance:**

In this qualitative study of administrator perspectives about admissions and care for individuals with OUD in SNFs, gaps in institutional capacity overlapped with stigmatizing beliefs about OUD; such beliefs perpetuate discrimination of individuals with OUD. Adequate SNF funding and staffing combined with OUD-specific interventions (eg, antistigma training, community partnerships for MOUD and recovery support) could incentivize SNFs to serve individuals with OUD and facilitate OUD care consistent with practice guidelines.

## Key Points

**Question:**

What do administrators perceive as barriers and facilitators to admitting and caring for residents with opioid use disorder (OUD) in skilled nursing facilities (SNFs)?

**Findings:**

This qualitative study included interviews with 29 SNF administrators in Rhode Island and described their perspectives on residents with OUD. Barriers included staffing and training deficits, reimbursement, regulatory oversight, and stigma; facilitators included transportation to methadone clinics or delivery of predosed methadone to SNFs, embedded psychiatric services, and coordination with social workers.

**Meaning:**

These findings suggest that there are opportunities for improvements in access to SNFs for persons with OUD and supports to incentivize facilities to provide such care.

## Teaser

This qualitative study analyzed skilled nursing facility (SNF) administrators’ perspectives on admissions and care for patients with opioid use disorder (OUD).

## Introduction

Rates of opioid use disorder (OUD) and overdoses have increased in nearly every age demographic in the US, with substantial increases among older adults.^1,2^ Along with the high mortality associated with opioid use, opioid-related acute care use and hospitalizations due to infectious complications (eg, endocarditis and skin infections) have become more frequent.^3,4,5^ Following hospitalization, individuals with OUD often require additional care to reduce the risk of complications, and skilled nursing facilities (SNFs) are an important location for managing subacute needs.^6^ One study^7^ estimated that 16% of hospitalized patients with OUD were discharged to SNFs; this finding may underestimate the need for SNF level of care in a population where the 30-day hospital readmission rate was nearly 30% among all OUD hospital discharges. Despite the growing relevance of SNFs for individuals with OUD,^8,9^ much remains unknown about admissions and care for residents with OUD in this setting.

Although hospitalized patients with OUD qualify for referral to SNFs at higher rates than patients without OUD, patients with OUD are substantially less likely to be accepted by SNFs,^10,11^ with some studies demonstrating rejection rates exceeding 80%.^12,13^ Thus, patients with a history of OUD experience prolonged hospitalizations while awaiting placement.^13,14,15^ The daily expense for an inpatient hospital stay is estimated at $2000, whereas SNF stays cost approximately one-fourth of this sum.^16^ With the shift toward value-based care in the US, there is an imperative to promptly transfer suitable patients from hospitals to SNFs. Despite federal antidiscrimination laws that protect individuals with OUD (unless they are engaged in active substance use) and those who are in treatment or recovery,^17^ an emerging literature^11,12,13,17,18,19,20,21^ suggests that individuals with OUD and those who receive medications for OUD (MOUD) are less likely to be placed in SNFs. Staff unpreparedness to care for residents with OUD; negative perceptions about risk to facility, staff, and other residents; lack of resources; and discriminatory admissions practices appear to limit access to SNFs.^19,20,21,22^ The existing literature is based on a few states, and, in some instances, emerged from expert opinion, pointing to the need for further empirical investigation in diverse states and more contemporary periods considering the evolving landscape of OUD treatment policies in the US.

Furthering understanding of the experiences and perspectives that shape the care of SNF residents with OUD can inform the development of tailored interventions and policy recommendations to enhance the quality of care for this underserved population. We conducted qualitative interviews with administrative and clinical leaders of SNFs (hereafter referred to as administrators) in Rhode Island to explore facilitators and barriers to accepting and caring for residents with OUD. By examining the perceptions of this group of stakeholders within SNFs, we intend to contribute insights that can guide efforts to optimize care and support for individuals with OUD in postacute and long-term care settings.

## Methods

### Study Design and Participants

This qualitative study was determined by the Brown University institutional review board to not to qualify as human participants research given that participants were interviewed in their professional capacity in accordance with the Common Rule. Independently, this study was approved by the Lifespan institutional review board. We obtained verbal consent. Written consent was waived because the study presented no more than minimal risk to participants. The study followed the Consolidated Criteria for Reporting Qualitative Research (COREQ) reporting guideline for reporting the study design, data analysis, and findings.^23^ We conducted semistructured interviews with administrators from SNFs in the US state of Rhode Island. Interviews occurred from November 5, 2021, to April 27, 2022. We recruited participants through direct outreach by telephone, email, and snowball sampling. For variation, sampling was conducted across the state independent of size and ownership status. We were interested in the general perception of caring for residents with OUD, including among administrators who may exclude this population from their facilities; therefore, we included participants who reported having no experience with OUD. Participants were compensated with a $100 gift card for their time.

### Study Procedures

We developed the interview guide (eAppendix in Supplement 1) with input from a person with OUD who had multiple experiences in SNFs, a researcher in long-term recovery, and Rhode Island’s long-term care ombudsman. Interviews lasted approximately 50 minutes. Questions were designed to capture perceptions, beliefs, and attitudes toward individuals with OUD or receiving MOUD while reconstructing the admissions process to better understand both barriers and successful strategies that exist in efforts to admit and care for these residents.

### Data Analysis

Audio files were transcribed utilizing Rev, an independent transcription service. The initial 5 interviews were coded line-by-line by 2 independent coders (S.N. and C.M.) to inductively generate candidate code books, which were then combined and refined through collective discussion. The remaining interviews were analyzed through code book thematic analysis and further independently coded by 2 coders (S.N. and C.M.) and reconciled in NVivo software version 1.7.1 (Lumivero). Following reconciliation, a senior research team member (J.S. or P.M.) reviewed each coded interview. Emergent codes were incorporated into the codebook through an iterative process. After 1 round of coding, the coding team met twice to distill themes based on a review of the entire process, including the analytical memos generated throughout. Themes were further revised through discussion with the larger research team and study advisors, and reanalysis of interviews was conducted based on these discussions. Data analysis occurred from August 22, 2022, to May 31, 2023.

We understand the inductive analysis of semistructured interviews as exploratory.^24^ Simultaneously, the incorporation of multiple perspectives into an iterative and reflexive coding process, including perspectives reflecting both clinical and lived experience, produce a more multidimensional exploration of the data than positivist approaches to saturation.

## Results

This qualitative study included 29 participants, (17 nursing home administrators [59%]; 6 directors of nursing [21%]; 5 directors of admissions [17%]; 1 unit manager [3%]), of whom 18 (62%) had worked 1 or more years in their role at the facility (Table 1). Participants were from 27 SNFs, representing nearly one-third of all SNFs in Rhode Island. Almost two-thirds of participants (18 participants) had experience with OUD referrals in their current role. We identified 4 main themes: (1) limited availability of mental health and addiction services; (2) staffing and industry crises; (3) concerns over resident population and facility culture; and (4) concerns about cost of care, funding, and regulatory oversight (Table 2). Additionally, we identified the ways that stigma was expressed in interviews as an additional theme that required a different level of analysis and posed specific methodological problems, which we address in the Discussion.

**Table 1.** Self-Reported Participant and Facility Characteristics

Characteristic	Participants or facilities, No. (%)
Participant characteristics (n = 29)	
Role in skilled nursing facility	
Administrator	17 (59)
Director of nursing	6 (21)
Director of admissions	5 (17)
Unit manager	1 (3)
Opioid use disorder and/or medications for opioid use disorder experience	
In current role	18 (62)
In previous role	9 (31)
None	2 (7)
Time in role at current skilled nursing facility, y	
<1	7 (24)
1-5	10 (34)
6-10	4 (14)
>10	4 (14)
Unknown	4 (14)
Facility characteristics (n = 27)	
Geographic area	
Rural	15 (56)
Urban or suburban	12 (44)
Facility ownership	
For-profit	20 (74)
Nonprofit	7 (26)
No. of beds, mean (range)	99 (33-220)

**Table 2.** Themes, Subthemes, and Illustrative Quotations in a Qualitative Study of Administrator Perspectives on Admission and Care for Residents With Opioid Use Disorder in Skilled Nursing Facilities

Theme and subtheme	Illustrative quotation
Limited availability of mental health and addiction services	
Logistics of providing medications for opioid use disorder	“So, when we get a referral with a resident that has the opiate use disorder, we look at the medical stuff first, of course, just to see if we are able to provide care for that particular resident. And then moving forward with the opioid use disorder, seeing if they’re on methadone, Suboxone; that makes a difference for us because we only have a contract with CODAC who will provide the methadone, but not the Suboxone. So, we have to see whether or not they’re registered with CODAC. And then if they are not, it’s a 30-day window that CODAC can supply that product. And then after that, they can’t continue to do so. So, that’s a little bit of a problem too. So, if we anticipate a stay to be longer than 30 days, we’re not able to take that resident in.” (Interview 7)
Community programming options	“So, help on that end and I would say also probably more services to come into buildings like NA, because a lot of times we had to transport people to AA or NA, and it being in a nursing home, staffing-wise is difficult.” (Interview 26)
Telehealth	“We do use [telehealth] occasionally here. Some of the residents don’t understand it because of their age. So, we have tried it; it doesn’t work 100%, but it’s good in a pinch.” (Interview 9)
Staffing and industry crises	
Staff shortages and turnover	“...half the people that work in nursing homes work there for one day and then they go work in a different one. And there’s not a lot of continuity of care when it comes to staffing these days. So, I mean, it’s not super consistent. So, you might train five people that might not ever be here again.” (Interview 27)
	“That’s our number one problem…I think what’s paramount right now, before they’re being given any consideration to take on a new patient or additional patients, the staffing issue has to be addressed. It’s a national issue, and it’s critical because we go day-to-day determining whether or not we can accept a new admission based on whether or not we have enough staff.” (Interview 6)
COVID-19	“So, our occupation rate has gone down about 20%, which is very devastating to our financial stature. People are actually keeping people home longer, and then just the devastation of having COVID-19 in a building. It did wipe out some population. And that hasn’t been replaced yet.” (Interview 26)
Concerns about resident population and facility culture	
Resident age	“So, I have a 29-year-old male in my building. I’m a 57-bed facility. And the majority of my residents are little, tiny old ladies. So, they have no one to talk to, they have no one who understands what they’re going through. They’ll end up staying in their room and being a little bit unsociable. And that’s a very hard thing for a person to have to go through.” (Interview 7)
	“Because of the current population we have here, is an elderly population who’s very vulnerable, that we’re afraid to mix the elderly population with young opiate addicted patients.” (Interview 9)
Behavior and social issues	“From our staff, they would be very concerned about it [admitting people with opioid addiction] because they have to deal with those residents one on one. And if there’s nobody there, those people could get violent, have outbursts, and things like that. So, it does become a concern.” (Interview 9)
	“Again, the staff not having the skill to care for it [OUD], no training, the potential for homelessness, would the patient become a discharge challenge? Same for us here. There’s certain social issues that would present more challenges than your typical skilled nursing patient.” (Interview 6)
Concerns about cost of care, funding, and regulatory oversight	
Liability	“No, but I do want to highlight how much it costs to take care of a younger person or somebody with substance abuse. Also, the liability issues, that worker’s compensation, injuries, injuries to staff, injuries to residents, incident reports. All of these things take time and take money.” (Interview 12)
Poor insurance reimbursement	“And then forget about it if you have Medicaid. They don’t even reimburse the facilities as much as it costs to care for them without IVs and therapy. The reimbursement, they don’t even pay us what we charge our private-pay patients per day. And that’s before any therapy or extra medications. So again, you have to look at who you’re taking in. You can’t always take the Medicaid patients, because you’re going to close.” (Interview 25)
Regulations and fines	“We’re cited through the Centers for Medicare and Medicaid. CMS will cite and it will directly affect our CMS five-star rating…so the rating will affect a lot of things. In Rhode Island, for example, the rating will affect certain insurance providers having contracts with the facility. So, theoretically, if you have a rating that’s poor, there are certain providers that will no longer keep you in-network. Your reimbursement will be hit. And if you fall so low, you can actually be fined from the state and from the feds.” (Interview 10)

Abbreviations: AA, Alcoholics Anonymous; CMS, Centers for Medicare and Medicaid; CODAC, CODAC Behavioral Healthcare; IV, intravenous; NA, Narcotics Anonymous; OUD, opioid use disorder.

### Theme 1: Limited Availability of Mental Health and Addiction Services

#### Logistics of Providing MOUD

Many facilities did not provide buprenorphine due to the lack of clinicians certified to prescribe the medication. Some participants reported having methadone delivered, which, though convenient, posed challenges regarding the timing of such deliveries and disposal of unused doses. Transporting residents to methadone clinics was cited as an additional expense not covered by existing funding, stretching already inadequate staff resources.

#### Community Programming Options

Participants stated that programs like Alcoholics Anonymous and Narcotics Anonymous would be helpful for residents with OUD, but transportation to meetings in the community was a barrier. Offering these programs or other programs (eg, individual therapy or facilitated groups) within the facility was also a reported concern given the lack of staff to support implementing such initiatives.

#### Telehealth

The COVID-19 pandemic increased the use of telehealth for mental health and addiction services in SNFs, though with mixed reviews. Some participants recognized that telehealth expanded access to services while others detailed staff burden in organizing calls multiple times each week and assisting residents such as those with schizophrenia or dementia. Positive experiences with the state’s 24/7 buprenorphine hotline for OUD treatment were described in limited interviews. Even among those who pointed to difficulties, participants emphasized that telehealth was necessary to offer some mental health and addiction support.

### Theme 2: Staffing and Industry Crises

#### Staffing Shortages and Turnover

The nationwide long-term care staffing crisis was consistently mentioned as a key obstacle to admitting and caring for residents with OUD. Participants shared that their inability to find staff led them to decrease occupancy or close entire SNF wings. Many participants emphasized gaps in continuity of care resulting from staffing constraints, given high staff turnover and reliance on temporary or per diem nurses. Participants perceived that OUD-specific training would be necessary for staff; therefore, the lack of staffing capacity sometimes became further rationale for deprioritizing OUD admissions.

#### COVID-19

The COVID-19 pandemic exacerbated staffing challenges and contributed to substantial declines in occupancy rates. Regulatory admission freezes and strategic admissions decisions such as (eg, extending admissions to individuals with OUD amid reduced occupancy rates) were also discussed.

### Theme 3: Concerns Over Resident Population and Facility Culture

#### Resident Composition

Often, participants viewed residents with OUD as younger, short-term residents, distinct from traditional older, long-term residents. Participants raised concerns about mixing these 2 populations. Some referenced resident activities and even food offerings as being geared toward their traditional residents, making it hard for a resident with OUD to fit in.

#### Behavior and Social Issues

In highly stigmatizing terms, some participants described a hypothetical person with OUD as violent, raising additional concern about housing them in the same room, or even the same building, as other residents. Past experiences attributed to residents with OUD included overdoses within the facility, bringing unapproved substances into the facility, theft, and undesirable visitors that might alarm family members of residents. These concerns left many participants worried about the facility’s liability if staff or other residents were injured or harmed.

### Theme 4: Concerns About Cost of Care, Funding, and Regulatory Oversight

#### Poor Insurance Reimbursement

There was a common perception of residents with OUD being more costly than other residents because of generally lower acuity among younger residents and low insurance reimbursement for residents with OUD who were overwhelmingly on Medicaid. Some participants stated that MOUD may not be reimbursed, creating costs to the facility. Participants expressed concerns that behavioral issues in residents with OUD may cause the facility’s ratings to decline, in turn affecting reimbursement rates.

#### Regulations and Fines

State regulations and fees associated with resident outcomes were also of concern to participants. Additionally, participants emphasized that if they decided not to readmit a resident (eg, following a self-directed discharge), or if they did not provide adequate documentation that the facility cannot care for a particular resident, they would get cited.

#### Liability

Participants frequently spoke about the existence of a high burden of proof placed upon SNFs by the state to establish that a facility truly cannot care for a resident safely. Participants described a sense of being under scrutiny by the state government, federal government, and the Alliance for Better Long-Term Care (the state’s ombudsman program).

### Theme 5: Stigma

#### Discrimination and Its Rationalization

Some participants described blanket bans on patients with OUD or receiving MOUD (Table 3). No participant described formal policy or admissions criteria for residents with OUD, but several described informal criteria used to evaluate admissions. The term stability was often used as a stand-in to reference these informal admissions criteria, yet participants were sometimes vague about what constituted that designation. In some interviews, receiving MOUD was seen as evidence of active addiction rather than treatment for OUD. This ambiguity and confusion resulted in the persistent blurring of clinical concerns and highly stigmatizing stereotypes.

**Table 3.** Subthemes and Illustrative Quotations for the Presentation of Stigma in the Perspectives of Skilled Nursing Facility Administrators

Subtheme	Illustrative quotation
Discrimination and its rationalization	“And unfortunately, we do know that typically, individuals who fall into this category [OUD] will do whatever it takes to get their fix. And, obviously, we do serve a population that is on schedule 2 hard narcotics. So, there are narcotics in the building. And that would be a concern for us, if this is a person who is at a high risk of relapse or has a history of relapsing and a history of violence, that would be a really big concern for us. We’re not a hospital. We don’t have 24-hour security onsite. We don’t have the ability to essentially take someone if they get out of hand.” (Interview 10)
	“We get most of them from the hospital. So just to give an idea. I just added up how many referrals we turned away for psych behaviors, alcohol, drug user, methadone, suicide, heavy smoker, and suboxone. We turned away 110 referrals this year.” (Interview 18)
Difficult question of stigma vs institutional capacity	“And, so, it’s almost like once they are admitted to you, they’re yours forever. And even like a short-term thing, say something really bad happened and you just said, ‘I can’t even assure their safety.’ They’re [the state] going to say, ‘Then you have to find them somewhere else to live.’ Which, again, is really unfair to the long-term care unit, because how are we going to find that? We’re not outside social workers.” (Interview 26)
	“Oh, yeah. So, I review it [the referral record], I’ll go through and weed out the obvious ‘no’s. So that would be one obvious no. If I see that they’re on methadone or Suboxone, I’m just like, ‘Nope, can’t do it.’” (Interview 25)

Abbreviation: OUD, opioid use disorder.

#### Difficult Question of Stigma vs Institutional Capacity

Contradictory attitudes toward residents with OUD emerged, combining genuine desire for resources to deliver better care and negative generalizations about OUD and mental illness. Participants often provided detailed descriptions of how past negative experiences in their facility reflected inadequate services and training, which interfered with caring for residents with OUD. Then, later in the interview, or sometimes even in the same passage, participants articulated stigmatizing generalizations to rationalize poor outcomes for this population.

## Discussion

This qualitative study elicited the perspectives of administrators on admissions and care for residents with OUD in SNFs. Several key findings emerged.

First, administrators pointed to persistent industry-wide challenges with funding and staffing^25,26,27^ as factors that hamper their capacity and willingness to invest in and innovate around care for residents with OUD. Administrators expressed hesitance to admit individuals with OUD due to the absence of existing behavioral health services within the facility. Logistical aspects of initiating, continuing, and managing MOUD in SNFs were viewed as a major consideration in admission decisions. While individuals with OUD or receiving MOUD were less likely to be admitted, participants also reported that bringing one’s own MOUD upon admission and availability of a medical director willing to initiate buprenorphine increased the likelihood of SNF admission. Some administrators reported leveraging community-based resources for OUD treatment to address institutional gaps in OUD care. Specifically, the delivery of predosed methadone to SNFs by CODAC (the largest provider of behavioral health care in Rhode Island) and telehealth through the state’s 24/7 buprenorphine hotline were noted as resources.^28^ The proposed federal Modernizing Opioid Treatment Access (MOTA) Act of 2023, which would allow methadone to be prescribed and dispensed outside certified opioid treatment programs, could enhance MOUD access in SNFs by allowing pharmacy-based methadone for OUD.^29,30^ Therefore, SNF residents stand to benefit from permanent methadone reforms that eliminate regulatory barriers to accessing treatment.^31^

Second, interviews identified concerns about the age of residents with OUD; these concerns were based on past experience and overlapped with stigma-driven concerns in many interviews, revealing a convoluted amalgam of attitudes. The majority of administrators pointed to difficulties of integrating younger residents into programming and institutional culture historically designed with much older residents in mind. Some administrators depicted residents with OUD (who often are younger residents) as violent and nonadherent, posing a threat to older adults who were considered traditional SNF residents. Importantly, this conflation of OUD with younger age risks overlooking the rapidly growing dangers of drug-related morbidity and mortality among older adults.^32,33,34^ Financial concerns were also a factor in age discrimination against younger people whose insurance reimbursements were perceived to be poor relative to older individuals who are mostly insured by Medicare.^27,35^ Inadequate funding and reimbursements are a substantial threat to SNFs, and facilities where the majority of residents rely on Medicaid are particularly at risk of closure.^36,37^ Therefore, financial incentives that encourage SNFs to admit younger people with Medicaid should be considered. Furthermore, the reduced occupancy rates in SNFs following the COVID-19 pandemic suggest that there may be business opportunities to extend admission to individuals perceived as nontraditional residents (ie, younger residents with OUD). For example, SNFs could establish specialized units designed to meet the needs of residents with OUD and other behavioral health needs. Some administrators considered such specialized units to have utility and likened them to memory care units, which are increasingly common.

Third, administrators commonly expressed seemingly self-contradicting views about reasons why residents with OUD may have poor experiences and outcomes. Administrators showed an understanding of *institutional mismatch*, a term we use to describe negative outcomes associated with residents with OUD being placed in facilities lacking the services, programming, or resources to fit their needs. We suggest the term *stigma ambivalence* to describe the ability of administrators to simultaneously recognize inadequate institutional capacity as a source of negative outcomes for residents with OUD, yet still redirect blame to individual residents for problematic behaviors and characteristics. In some cases, participants voiced ambivalence by noting that they should not generalize about groups but still holding that these generalizations held true. Even when participants did not express explicit stigma ambivalence, the uneasy interplay between 2 modes of explanation—one based on experience and the other filtered through stereotype—created a tension that differed from interviews characterized by more straightforward assertions of stigma. Stigma ambivalence made it impossible, in many cases, to distinguish between genuine concerns over possessing institutional capacity and stereotyping of individuals with OUD. Of note, some aspects of stigma presented in discriminatory practices (eg, blanket admission bans and rejecting individuals for receiving MOUD), which are expressly prohibited by protections for individuals with OUD under the American Disabilities Act (ADA).^17^ Despite challenging market forces, SNFs still need to comply with the protections for people with OUD or receiving MOUD afforded by the ADA. Capitalizing on the elimination of the buprenorphine X-waiver requirement and extended methadone take-home flexibilities could help reduce discrimination against individuals with OUD and build onramps to OUD treatment in SNFs.^46,47,48^ If passed, the MOTA Act would further reduce barriers to accessing MOUD in SNFs by allowing pharmacy dispensing of methadone for OUD.

While administrators recognized possible actions to remedy gaps in institutional capacity to provide enhanced care quality for residents with OUD, some administrators hesitated to take such actions to avoid attracting nontraditional residents. In this context, only in limited instances might SNFs voluntarily train staff or bolster infrastructure to support residents with OUD. Therefore, meaningful advancements toward accessible, evidence-based SNF care for individuals with OUD are unlikely without broader federal and state investment dedicated for this purpose.^38^ Beyond financial incentives, such as increased Medicaid reimbursement, there is need for greater investment in existing or new partnerships to enhance stigma reduction and improve SNF relations with accountability structures. While more regulations are often not the answer, nuanced considerations of minimum standards of behavioral health services consistent with the level of care provided in SNFs are warranted.^18,39^

### Limitations

This study has limitations. Interpretation of our findings should account for attitudinal fallacy, the unfounded inference that views expressed in a single setting are undistorted indicators of real-world behaviors.^40^ More empirical work is required to reconstruct the dynamics of the admission process, including unconscious biases or views minimized due to social acceptability bias. It is noteworthy that a minority of participants were directly involved in patient care. The views of administrators may differ in important ways than direct care staff and others (eg, hospital discharge planners and social workers) who were not observed. Although semistructured interviews do not allow for a controlled comparison regarding attitudes toward different conditions, our interviews revealed that administrators frequently conflated people with OUD with other residents that placed strain on their facility in terms of cost, staffing, and culture. This finding suggests that our OUD-specific framing may capture only part of the pressures and constraints that informed their responses.

Our interviews may have been influenced by preceding judgements against SNFs for discrimination against people with OUD in the neighboring state of Massachusetts based on the expanded guidelines for OUD under the ADA.^12^ Simultaneously, our study took place before affirmations of antidiscrimination protections became more widely publicized across the country, which may have allowed administrators to give voice to views that they would be more cautious in openly expressing today.^41,42^ Finally, qualitative research among individuals with OUD with lived experience in SNFs deserves exploration in future research because these perspectives have not previously been described. They are essential, not only to design patient-centered OUD services in SNFs, but also to provide critical context to interpret the views expressed by SNF administrators. Future research exploring the intersectionality of OUD with other identities (eg, race, ethnicity, sex, and gender identity) is also warranted to promote equity in SNF access.^43,44,45^

Notwithstanding these limitations, our study makes novel contributions by elucidating the logistical challenges and potential solutions to providing MOUD in SNFs. Further, to our knowledge, stigma ambivalence has not been previously described in the literature on stigma and substance use disorders; therefore, naming this pattern adds novelty to the literature.

## Conclusions

This qualitative study among SNF administrators found that being diagnosed with OUD introduced challenges to accessing SNFs and that opportunities to initiate or continue MOUD were inconsistent and often nonexistent across facilities. Administrators often expressed resource, training, or facility capacity concerns, yet these concerns overlapped with stigmatizing beliefs about OUD and prohibited discriminatory practices. Additionally, addressing longstanding SNF issues (eg, funding and staffing) and creating OUD-specific interventions (eg, antistigma training, community partnerships for MOUD, and recovery support) could facilitate SNF care for OUD that is consistent with practice guidelines.

## Notes

Supplement 1. **eAppendix.** Interview Guide Used to Conduct the Qualitative Study of Administrator Perspectives on Admissions and Care for Individuals With Opioid Use Disorder in Skilled Nursing Facilities

Supplement 2.
Data Sharing Statement
