# Supplementary material for: Perspectives on Admissions and Care for Residents With Opioid Use Disorder in Skilled Nursing Facilities
Source: JAMA Netw Open. 2024 Feb 5;7(2):e2354746. doi: 10.1001/jamanetworkopen.2023.54746 (PMC10844991; doi:10.1001/jamanetworkopen.2023.54746)
Supplement: Supplement 1. — eAppendix. Interview Guide Used to Conduct the Qualitative Study of Administrator Perspectives on Admissions and Care for Individuals With Opioid Use Disorder in Skilled Nursing Facilities [file jamanetwopen-e2354746-s001.pdf]

Supplemental Online Content

Moyo P, Nishar S, Merrick, C. Perspectives on admissions and care for residents with opioid use disorder in skilled nursing facilities. *JAMA Netw Open*. 2024;7(2):e2354746. doi:10.1001/jamanetworkopen.2023.54746

eAppendix. Interview Guide Used to Conduct the Qualitative Study of Administrator Perspectives on Admissions and Care for Individuals With Opioid Use Disorder in Skilled Nursing Facilities

This supplemental material has been provided by the authors to give readers additional information about their work.

© 2024 Moyo P et al. *JAMA Network Open.*

eAppendix. Interview Guide Used to Conduct the Qualitative Study of Administrator Perspectives on Admissions and Care for Individuals With Opioid Use Disorder in Skilled Nursing Facilities

Introduction

- ❑ Hi, **[insert participant name]**, how are you doing? Thank you for making time to participate in our study.
- ❑ My name is **[insert name]**; I am one of the research assistants working on this study.
- ❑ Is it okay if I record this interview? The recording will be used only for research purposes.
- ❑ In this study, we are trying to understand some of the issues that come up when people with opioid addictions (Opioid Use Disorder) are referred to skilled nursing facilities.
- ❑ This interview will have two parts. Each will last roughly 30 minutes.
- ❑ First, I will ask you some open-ended questions about your experience as a SNF administrator, and if you've never dealt with people with opioid use disorder directly, what you think some of the challenges might be.
- ❑ Second, I will ask you more structured questions about your facility and its policies.
- ❑ If you would like to stop at any time, just let me know. You don't need to answer any question that you don't want to and, remember, all of your answers will be kept completely confidential.
- ❑ Thank you again for previously consenting to participate in this study.

PART 1

Question 1: Before we get started, will you tell me a little about your background and professional experience working in skilled nursing facilities?

Question 2: Have you ever dealt with a case of a person with opioid addiction or receiving medication for an opioid addiction such as buprenorphine (also known as Suboxone) or methadone who was trying to enter a skilled nursing facility?

[If the answer is "yes", proceed to section A. If the answer is no, by-pass section A completely and start with section B]

© 2024 Moyo P et al. *JAMA Network Open.*

SECTION A

Question 3a: We would like to get a general or "big picture" idea of all of the issues involved. Let me first ask: how many cases of opioid use disorder have you have dealt with in your current role?

[If the answer is more than one, proceed to ask questions 4a and 5a immediately below under *Multiple Cases* If the answer is 1, skip *Multiple Cases* and proceed to answer question 4a under *Single Case*]

Multiple Cases

Question 4a: Would you start by walking me through the most straightforward case, telling me what you remember of the admissions process and the resident's time at the facility? Please start at the beginning and walk me through step-by-step.

Question 5a: Thank you. Now, could we discuss a more involved or complicated case? Let's go through it the same way. Start at the beginning and walk me through the admission process and the resident's time at the facility.

Thank You. I am now going to ask you some more specific questions about the experience of your facility with people with opioid addiction. **[PROCEED to Question 6a]**

Single Case

Question 4a: Would you walk me through the case, telling me what you remember of the admissions process and the residents' time at the facility? Start at the beginning and walk me through step-by-step.

Thank You. I am now going to ask you some more specific questions about this case. **[PROCEED to Question 6a.]**

Question 6a: I would like to talk through this case/these cases in more detail. Let's start from the beginning. After the facility got the referral, who made the admission decision/s?

Question 7a: Does your facility make use of a psychiatric evaluation or service? **[If the answer if yes, ask next question]** What role does it play during the admission process of someone with opioid addiction?

Question 8a: Looking back at this case/these cases, what do you remember as going well with the resident's/residents' time at the facility? What were the successes? What do could have gone better?

Question 9a: During their stay, were there challenges or problems that you didn't anticipate?

Question 10a: When you were talking about this admission/these admissions, did any issues come up that were different than an ordinary admission?

Question 11a: Did anyone raise concerns about admitting someone with an opioid addiction to your facility? **[Follow up questions: what were the concerns?]**

© 2024 Moyo P et al. *JAMA Network Open.*

Question 12a: Do you remember if those concerns were based on previous experiences at your facility, things people heard from other facilities, or personal experiences?

Question 13a: Were there any concerns about how staff or residents might feel about admitting the resident/the resident?

Question 14a: How did the age of a person with opioid addiction influence the individuals'/individual's experience in the SNF?

Question 15a: Thinking back on this case, were there resources that you didn't have, but would have been useful in caring for the resident?

Question 16a: Do you remember if the resident received any extra support or monitoring for their opioid addiction? **[If they ask for clarification, add "Did they receive additional therapy or transport to support groups like Narcotics Anonymous?"]**

Question 17a: What additional services are available at your facility for people who need support for addictions? [such as AA/ NA support groups]

Question 18a: Do you remember if the patient was on Suboxone (also known as buprenorphine) or methadone?

[If the answer is "yes", clarify which medication. If the medication is suboxone, follow up with: Do you remember if there were problems with getting suboxone from the pharmacy for the resident? Were there any other challenges related to the resident being on suboxone?]

Question 19a: How are decisions regarding the discharge of people with opioid addiction from SNF made? Have you had cases where the individual was homeless or unstably housed?

Question 20a: How do you think the experience of your facility with this case/these cases compares with other facilities?

Question 21a: Is there anything that I did not ask about that our research team should know about people with opioid addictions either entering or living in skilled nursing facilities?

SECTION B: Hypothetical Case

Question 3b: No worries. We would like to get a general or "big picture" idea of all of the issues involved, so I would like to ask you some hypothetical questions. Could you start by just walking us through the admission process for a person with opioid addiction or on a medication such as suboxone or methadone for an opioid addiction?

Question 4b: Thank you. Now, I would like to ask some more specific questions. Let's start from the beginning. After the facility gets the referral, who would make the admission decision?

© 2024 Moyo P et al. *JAMA Network Open.*

Question 5b: Does your facility make use of a psychiatric evaluation or service? **[If the answer is yes, ask the next question]** What role would it play during the admission process of someone with opioid addiction?

Question 6b: Do you think that there would be any questions or concerns in admitting a person with opioid addiction (whether or not they are on medication for opioid addiction) that are different than a normal admission?

Question 7b: How do you think that staff and residents would respond to the admission of a person with opioid addiction or receiving treatment for this condition?

Question 8b: Would your facility be able to offer any extra supports or services aimed at treating their opioid addiction? **[Examples: additional therapy or transport to support groups like Narcotics Anonymous or to methadone clinics.]**

Question 9b: Would you foresee any challenges specific to residents receiving medications for opioid addiction? **[If they ask for clarifications, you could ask if there are concerns about pain management billing and reimbursement, ability to initiate or continue prescription for medication for opioid addiction, logistical and regulatory challenges.]**

Question 10b: How would the age of a person with opioid addiction influence the individual's time in the facility?

Question 11b: Are there resources that you don't currently have that you think would be useful for caring residents on medications for opioid use disorder?

Question 12b: Have you heard about these types of cases in other facilities? **[Follow-up if yes: what have you heard about these cases?]**

Question 13b: How are decisions regarding the discharge of people with opioid addiction from SNF made? Have you had cases where the individual was homeless or unstably housed?

Question 14b: Is there anything that I did not ask about that our research team should know about issues relating to people with opioid addictions either entering or living in skilled nursing facilities?

© 2024 Moyo P et al. *JAMA Network Open.*

PART 2

Now I would like to ask you some more specific questions about your facility and its policies.

1. Do you perceive that there is increasing demand for post-acute and long-term care for people with opioid or other addictions?

2. Have you ever received any education/training about Suboxone (also known as buprenorphine), methadone, or medications for opioid use disorder in general? [If not, where has your knowledge come from? **Examples:** media, co-workers, personal acquaintances]

3. Have you ever had a patient on methadone? What are the logistics of methadone delivery and storage at your SNF?

4. Are there any formal or informal guidelines at your facility related to admission and/or management of residents with addictions?
   a) **[If yes]** Can you tell me more about these guidelines? **[After the answer is complete: ask if you can have a copy of the guidelines]**

5. Does your facility currently have guidelines in place regarding the treatment of residents with opioid addiction utilizing Suboxone (also known as buprenorphine) or methadone?
   a) **[If no]** Are there any informal policies or guidelines in place?
   b) Is the pharmacy able to stock and dispense Suboxone?

6. Would concerns about managing pain with opioids affect the decision to admit a person with opioid addiction into your facility?

7. Does your facility have policies related to addiction, substance misuse, or supporting residents in recovery?

8. What resources or treatments have you seen help residents with alcohol or substance use problems, including problems other than opioid addictions?

9. Do you think special programs/initiatives would improve access to SNFs by patients receiving medication for opioid addiction?

10. Do you *agree, disagree* or are you *neutral* about the **usefulness** of implementing each of the following programs in SNFs?:
    a) Formal referral relationship between SNFs and external providers to prescribe or dispense medication for opioid addiction.
    b) Training for staff in SNFs to prescribe and dispense medication for opioid addiction.
    c) Behavioral health care access in SNFs (e.g., using telehealth for appointments with trained and qualified mental health and addiction professionals).
    d) Delivery of medication for opioid addiction to SNFs by an outreach program such as CODAC. [**If needed, explain that CODAC Behavioral Healthcare is the largest non-profit organization in Rhode Island that provides outpatient treatment for opioid addiction**]

11. Do you *agree, disagree* or are you *neutral* that it is **feasible** to implement the following programs in SNFs?:

© 2024 Moyo P et al. *JAMA Network Open.*

a) Formal referral relationship between SNFs and external providers to prescribe or dispense medication for opioid addiction.
b) Training for staff in SNFs to prescribe and dispense medication for opioid addiction.
c) Behavioral health care access in SNFs (e.g., using telehealth for appointments with trained and qualified mental health and addiction professionals).
d) Delivery of medication for opioid addiction to SNFs by an outreach program such as CODAC.

Demographic Questions

Now, I will ask you some basic questions about your background and the facility type

Interviewee background

1. What is your Position/Title at [SNF]?
2. How long have you been in this role: in years?
3. How many years of total clinical/professional experiences do you have (including time spent in other facilities/places of employment)?
4. Can you tell me about your education attainment (e.g., Bachelor's degrees, associate degree, Master's degree or higher)? **[If not already described earlier as part of background]**

About the facility where participant works

5. Is your SNF a non-profit or for-profit facility. [**If other, ask about what the SNF ownership type is. For example, government-owned, religious, private etc.]**
6. Would you describe the SNF location as urban or rural?
7. What is the maximum number of residents in this facility?
8. Has the COVID-19 pandemic affected occupancy rates or aspects of the resident population in your SNF?

Conclusion

- ❑ Again, thank you for taking time to participate in this interview and for sharing with us.
- ❑ We will be in touch to provide you with an Amazon gift card. We plan to send the gift card via email. What email address would you like us to send the e-gift card to?
- ❑ Are you willing to be contacted for a follow-up interview if needed?
- ❑ Also, if you have any questions or any suggestions for us, please feel free to contact me at **[inset email]** or by phone on at **[phone number]**.

© 2024 Moyo P et al. *JAMA Network Open.*
