# Supplementary material for: Perspectives on Admissions and Care for Residents With Opioid Use Disorder in Skilled Nursing Facilities
Source: JAMA Netw Open. 2024 Feb 5;7(2):e2354746. doi: 10.1001/jamanetworkopen.2023.54746 (PMC10844991; doi:10.1001/jamanetworkopen.2023.54746)
Supplement: Supplement 2. — Data Sharing Statement [file jamanetwopen-e2354746-s002.pdf]

Data Sharing Statement

Moyo. Perspectives on Admissions and Care for Residents With Opioid Use Disorder in Skilled Nursing Facilities. *JAMA Netw Open*. Published February 05, 2024.
doi:10.1001/jamanetworkopen.2023.54746

Data
Data available: No

Additional Information
Explanation for why data not available: The interview guide used in this study has been provided as an online supplement. In accordance with the consent form used for this study, audio recordings will be destroyed at the completion of the study and therefore cannot be shared. Researchers interested in deidentified interview transcript data should contact the corresponding author (Email: patience_moyodow@brown.edu).
